# Patterns of variation of mutation rates of mitochondrial and nuclear genes of gastropods

**DOI:** 10.1186/s12862-021-01748-2

**Published:** 2021-01-26

**Authors:** Thomas F. Duda

**Affiliations:** Museum of Zoology & Department of Ecology of Evolutionary Biology, University of Michigan, 1105 N. University, Ann Arbor, MI 48109-1085 USA

**Keywords:** Gastropoda, Mutation rates, Mitochondrial DNA, Nuclear DNA

## Abstract

**Background:**

Although mitochondrial DNA (mtDNA) of many animals tends to mutate at higher rates than nuclear DNA (nuDNA), a recent survey of mutation rates of various animal groups found that the gastropod family Bradybaenidae (suborder Helicina) shows a nearly 40-fold difference in mutation rates of mtDNA ($\mu$_m_) and nuDNA ($\mu$_n_), while other gastropod taxa exhibit only two to five-fold differences. To determine if Bradybaenidae represents an outlier within Gastropoda, I compared estimated values of $\mu$_m_/$\mu$_n_ of additional gastropod groups. In particular, I reconstructed mtDNA and nuDNA gene trees of 121 datasets that include members of various clades contained within the gastropod subclasses Caenogastropoda, Heterobranchia, Patellogastropoda, and Vetigastropoda and then used total branch length estimates of these gene trees to infer $\mu$_m_/$\mu$_n_.

**Results:**

Estimated values of $\mu$_m_/$\mu$_n_ range from 1.4 to 91.9. Datasets that exhibit relatively large values of $\mu$_m_/$\mu$_n_ (i.e., > 20), however, show relatively lower estimates of $\mu$_n_ (and not elevated $\mu$_m_) in comparison to groups with lower values. These datasets also tend to contain sequences of recently diverged species. In addition, datasets with low levels of phylogenetic breadth (i.e., contain members of single genera or families) exhibit higher values of $\mu$_m_/$\mu$_n_ than those with high levels (i.e., those that contain representatives of single superfamilies or higher taxonomic ranks).

**Conclusions:**

Gastropods exhibit considerable variation in estimates of $\mu$_m_/$\mu$_n_. Large values of $\mu$_m_/$\mu$_n_ that have been calculated for Bradybaenidae and other gastropod taxa may be overestimated due to possible sampling artifacts or processes that depress estimates of total molecular divergence of nuDNA in groups that recently diversified.

## Background

Information concerning patterns of variation of mutation rates of nuclear and organellar genomes is important for understanding the factors that influence these rates and how they impact interactions among genomes [1–4]. For most animal taxa, mutation rates ($\mu$_m_) of mitochondrial DNA sequences (mtDNA) are higher than mutation rates ($\mu$_n_) of nuclear DNA sequences (nuDNA) [5–7]. Nonetheless, invertebrates tend to show smaller differences between $\mu$_m_ and $\mu$_n_ (~ 2 to 10-fold difference) than vertebrates (~ 10 to 25-fold difference) [3, 4, 7–11]. A recent comparison of estimates of the ratio $\mu$_m_/$\mu$_n_ of 122 animal taxa (one sponge, 78 vertebrates, 33 arthropods, and 10 molluscs) found that one mollusc group, members of the gastropod family Bradybaenidae (subclass Heterobranchia, order Stylommatophora, suborder Helicina), shows a nearly 40-fold difference in $\mu$_m_ and $\mu$_n_, whereas other molluscs, including representatives of six other gastropod groups, exhibited only two to five-fold differences [4]. Is Bradybaenidae an exception within Mollusca or do other gastropods (e.g., other members of the species rich suborder Helicina) have exceptionally large values of $\mu$_m_/$\mu$_n_? Furthermore, what potential factors contribute to the large values of $\mu$_m_/$\mu$_n_ that Bradybaenidae and possibly other gastropod groups exhibit?

To address the above questions, I estimated and compared $\mu$_m_/$\mu$_n_ of additional gastropod groups, including members of four of the six subclasses of Gastropoda—Caenogastropoda, Heterobranchia, Patellogastropoda, and Vetigastropoda—and additional groups from the suborder Helicina. I aimed to determine if Bradybaenidae is an outlier within Gastropoda or if other gastropod taxa also exhibit such large values of $\mu$_m_/$\mu$_n_. I also evaluated whether variation in $\mu$_m_/$\mu$_n_ among gastropod taxa reflects relative differences in $\mu$_m_ or $\mu$_n_ among groups exhibiting different values of $\mu$_m_/$\mu$_n_. In addition, I sought to determine if large values of $\mu$_m_/$\mu$_n_ might be overestimated due to possible inclusion of recently diverged species by comparing ratios of $\mu$_m_/$\mu$_n_ from datasets that include different levels of taxonomic breadth (i.e., those that include members of genera, families, superfamilies, or higher taxonomic categories). I largely followed the approach described in [4] of gathering published mtDNA and nuDNA gene sequences from corresponding sets of individuals/species, reconstructing gene trees, and then inferring relationship between $\mu$_m_ and $\mu$_n_ by calculating the ratio of the total branch lengths of mtDNA and nuDNA gene trees. I estimated total branch lengths at third codon positions for coding regions (as implemented in [4]) and all positions of intron regions as a proxy for neutral divergence.

## Results

### Datasets

Searching for the term “Gastropoda [Organism]” in the NCBI PopSets database yielded 3692 datasets. More than two-thirds of the PopSets (N = 2593) contained mtDNA sequences and more than half of these (N = 1551) included COI sequences (Table 1). Most nuDNA sequences included sequences of various regions of the rRNA transcription unit (i.e., 5.8S, 18S, 28S, and internal transcribed spacers 1 and 2 (ITS1 and ITS2)) (N = 639) (Table 1). Otherwise, sequences of histone (mostly histone H3 (H3)) comprised the next most abundant nuDNA gene region included in these PopSets (Table 1).

**Table 1:** Identity of mtDNA and nuDNA gene regions that were included in the 3692 NCBI PopSets

Gene	Number
Mitochondrial genes	2593
12S	120
16S	784
cytb	79
COI	1551
COII	13
NADH	30
Other genes	124
Nuclear genes	1099
rRNA (5.8S, 18S, 28S, ITS1, ITS2)	639
Actin	19
Conotoxins	28
EF1$\alpha$	12
Histone (H3, H4)	215
Other genes	186

Only gene regions included in 10 or more PopSets are listed

Cytb: cytochrome *b*; COI: cytochrome oxidase subunit I; COII: cytochrome oxidase subunit II; NADH: nicotinamide adenine dinucleotide; ITS1: internal transcribed space I; ITS2: internal transcribed space 2; EF1$\alpha$: elongation factor 1-alpha

I identified 118 sets of PopSets from the same author(s) that included both mtDNA and nuDNA data of protein-coding regions or introns of at least three species. All but four of the mtDNA sequence datasets included COI sequences; the four exceptions contained cytochrome *b* (cytb) sequences. All but six of the nuDNA sequence dataset included coding regions of histone H3 (H3); these other datasets contained coding regions of actin, adenine nucleotide translocase (ANT), histone H4 (H4; two datasets), and a megalin-like lipoprotein (mlp) gene as well as sequences of an intron of a gamma glutamyl carboxylase gene.

Five sets of sequences included data from two mtDNA genes and one nuDNA gene (N = 2; COI and cytb in both cases) or from two nuDNA genes and one mtDNA gene (N = 3; H3 and H4 in two cases and H3 and ANT in the other). I also included datasets of the two sets of mtDNA and nuDNA that were previously examined in [4] but not uploaded originally to NCBI as PopSets (i.e., COI and H3 sequences of Bradybaenidae and Aglajidae from [12] 12]). I divided mtDNA and nuDNA sequences of one set of PopSets into three sets of individual alignments that each contained sequences of single superfamilies of the subclass Vetigastropoda (i.e., Fissurelloidea and Lepetelloidea from the order Lepetellida, and Trochoidea from the order Trochida); this was performed to enable comparison of $\mu$_m_/$\mu$_n_ from these lower taxonomic categories. I also combined two sets of PopSets from the same author(s) given that the different PopSets included sequences from members of the same family. Hence, in total I examined 121 sets of mtDNA and nuDNA data. The datasets contain sequences of members of four gastropod subclasses, including Caenogastropoda (N = 31), Heterobranchia (N = 81), Patellogastropoda (N = 1), and Vetigastropoda (N = 8), as well as a considerable breadth of the superfamilies (N = 34) contained within these clades (Additional file 1: Table S1). While most datasets contained representatives of single genera (N = 54), others included members of single families (N = 34), superfamilies (N = 18), or higher level ranks (N = 15).

### Sequence analyses

I reconstructed mtDNA and nuDNA gene trees using maximum likelihood approaches and then calculated total branch lengths (TBL) of these trees at third positions of codons of coding regions or all positions of intron sequences with MEGAX v.10.1.8 [14]. I then calculated the ratio of these values (i.e., $\mu$_m_/$\mu$_n_) for PopSet pairs that included the same species (Additional file 1: Table S1).

**Fig. 1:**
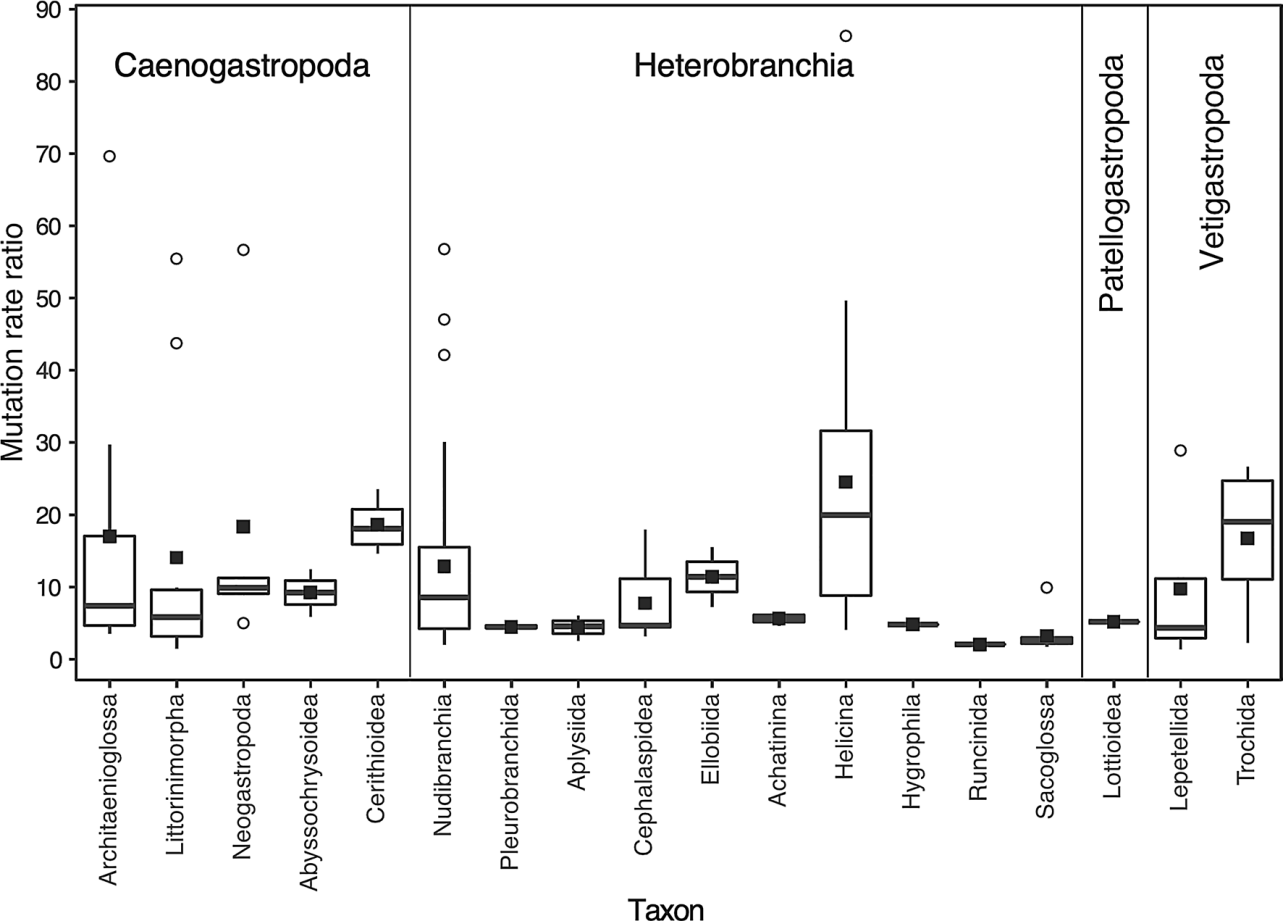
Boxplots of mutation rate ratios ($\mu$_m_/$\mu$_n_) of datasets of members of gastropod clades from the subclasses Caenogastropoda, Heterobranchia, Patellogastropoda, and Vetigastropoda. Median values (wide horizontal line in box of taxa with more than one dataset), interquartile ranges (boxes), minimum and maximum range (ends of whiskers that extend 1.5 times the interquartile range), means (solid square symbols), and outlier values (open circle symbols) are indicated

Estimates of $\mu$_m_/$\mu$_n_ of individual datasets ranged from a minimum of 1.4 (Vetigastropoda; Lepetellida; Fissurelloidea) to a maximum of 91.9 (Heterobranchia; Tectipleura; Helicina; Clausilioidea; Clausiliidae) with most taxa showing mean ratios that are less than 20 and median ratios that are less than ten (Fig. 1, Additional file 1: Table S1). Gene trees of nuDNA sequences of two PopSets exhibited a TBL of zero (PopSet UID 1735180796, genus *Viviparus*, family Viviparidae, order Architaenioglossa; PopSet UID 1125137272, genus *Acroloxus*, family Acroloxidae, superorder Hygrophila); results from analyses of these datasets were excluded from those that utilize ratios of $\mu$_m_/$\mu$_n_ or log-transformed values of TBL of nuDNA given that these values are undefined.

Results from an ANOVA that compared $\mu$_m_/$\mu$_n_ values of datasets from members of higher level taxonomic groups (i.e., superorders, orders, and suborders) revealed significant differences in $\mu$_m_/$\mu$_n_ among these groups (P = 0.00137). Based on results from a Tukey test, mean values of $\mu$_m_/$\mu$_n_ are significantly different among Helicina (20.1) and Sacoglossa (3.3) and Cerithioidea (18.6) and Sacoglossa (Fig. 1). In addition, several groups include datasets that exhibit values of $\mu$_m_/$\mu$_n_ that represent outliers in boxplots (Fig. 1). These include datasets from Architaenioglossa, Littorinimorpha, and Neogastropoda in Caenogastropoda; Nudibranchia, Helicina, and Sacoglossa in Heterobranchia; and Lepetellida in Vetigastropoda (Fig. 1, Table 2).

**Table 2:** Datasets that exhibit outlier values of $\mu$_m_/$\mu$_n_ (Fig. 1)

Group	Lowest taxon	$\mu$_m_/$\mu$_n_	Gene regions (mtDNA/nuDNA)	PopSets
Architaenioglossa	*Bellamya* (Viviparidae)	69.6	COI/H3	515025847, 515025915
Littorinimorpha	*Asterophila* (Eulimidae)	43.7	COI/H3	1720684427, 1720684543
Littorinimorpha	*Naticarius* (Naticidae)	55.5	COI/H3	239836736, 239836785
Neogastropoda	*Conus* (Conidae)	56.7	cytb/mlp	57648245, 62632959
Neogastropoda	Conoidea	5.9	COI/H3	160279556, 160279786
Nudibranchia	*Chromodoris* (Chromodorididae)	42.1^a^	COI/H3 + ANT	1352800289, 1352800309, 1352800449
Nudibranchia	*Chromodoris* (Chromodorididae)	56.8	COI/H3	1693116299, 1693116247
Nudibranchia	*Doriopsilla* (Dendrodorididae)	47.0	COI/H3	924919646, 924919558
Helicina	*Cristitaria* (Clausillidae)	86.6^a^	COI/H3 + H4	1610157399, 1610157415, 1610157433
Sacoglossa	*Placida* (Limapontiidae)	9.9	COI/H3	1386665768, 1386665790
Lepetellida	*Haliotis* (Haliotidae)	28.9	cytb/H3	1569125434, 1569125484

The lowest taxon that encompasses the majority of the species included in PopSets is indicated; families of genera also indicated

ANT: adenine nucleotide transferase; cytb: cytochrome *b*; COI: cytochrome oxidase subunit I; mlp: megalin-like lipoprotein

^a^Average $\mu$_m_/$\mu$_n_ based on two nuDNA gene regions

**Fig. 2:**
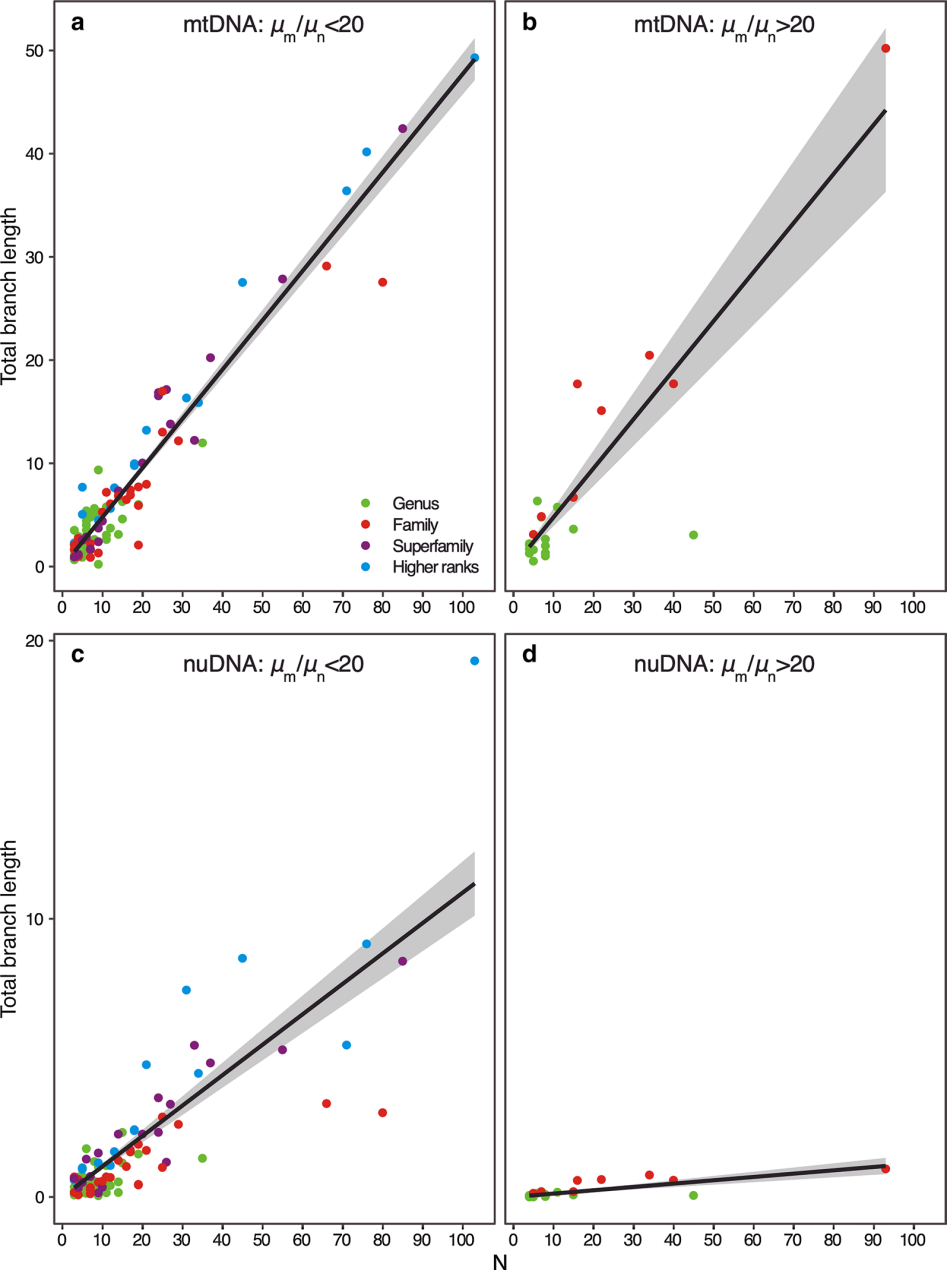
Relationships between number of species and total branch lengths of gene trees. **a** mtDNA datasets with $\mu$_m_/$\mu$_n_ < 20, R^2^ = 0.934, P < 2.2 × 10^–16^; **b** mtDNA datasets with $\mu$_m_/$\mu$_n_ > 20, R^2^ = 0.778, P < 7.8 × 10^–8^; **c** nuDNA datasets with $\mu$_m_/$\mu$_n_ < 20, R^2^ = 0.705, P < 2.2 × 10^–16^; **d** nuDNA datasets with $\mu$_m_/$\mu$_n_ > 20, R^2^ = 0.652, P < 5.8 × 10^–6^

The number of species (N) included in mtDNA and nuDNA gene trees and TBL of these trees exhibit strong positive associations (Fig. 2). Relationships between N and TBL of mtDNA gene trees are not significantly different for datasets with relatively low values of $\mu$_m_/$\mu$_n_ (i.e., < 20) and those with relatively high values (i.e., > 20) (P = 0.643) (Fig. 2a, b). On the other hand, relationships among N and TBL are significantly different for nuDNA data from low ratio and high ratio datasets (P < 2.2 × 10^–16^), with TBL of high ratio datasets exhibiting a much lower rate of increase with increasing N in comparison to low ratio datasets (Fig. 2c, d).

**Fig. 3:**
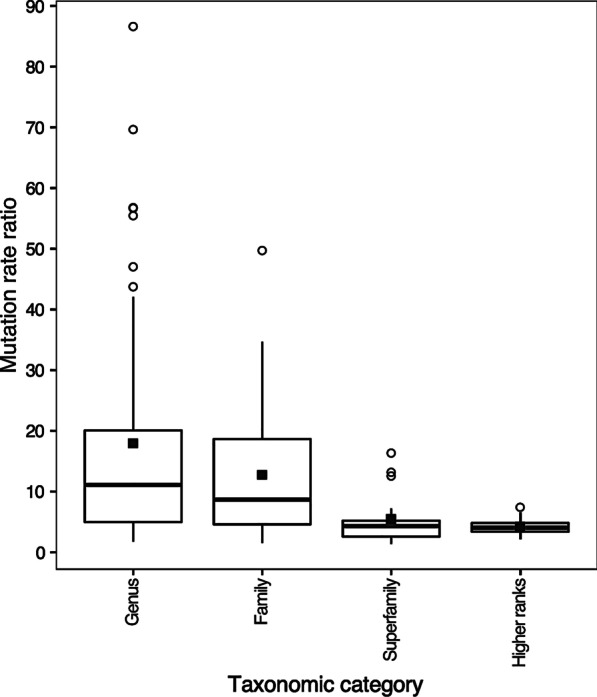
Boxplots of mutation rate ratios ($\mu$_m_/$\mu$_n_) of datasets that include different levels of taxonomic breadth (i.e., genera, families, superfamilies, and high taxonomic ranks). Median values (wide horizontal line in box), interquartile ranges (boxes), minimum and maximum range (ends of whiskers that extend 1.5 times the interquartile range), means (solid square symbols), and outlier values (open circle symbols) are indicated

Datasets that include representatives of genera and families exhibit significantly different values of $\mu$_m_/$\mu$_n_ based on ANOVA and Tukey tests (P = 0.000055). In particular, while the mean $\mu$_m_/$\mu$_n_ of datasets that contained members of genera and families were 17.9 (SD = 19.3, N = 52) and 12.7 (SD = 11.4, N = 34), respectively, mean values of $\mu$_m_/$\mu$_n_ of datasets that included members of superfamilies (5.5, SD = 4.2, N = 19) and higher taxonomic categories (4.2, SD = 4.2, N = 15) were lower (Fig. 3). Also, although all four categories exhibit outliers in boxplots, only outliers from genera and families exhibited values of $\mu$_m_/$\mu$_n_ that were greater than 20 (Fig. 3).

## Discussion

A previous survey that examined relationships of $\mu$ of mtDNA and nuDNA of 122 animal taxa found that the gastropod family Bradybaenidae (subclass Heterobranchia, order Stylommatophora, suborder Helicina) exhibits a nearly 40-fold difference in $\mu$_m_ and $\mu$_n_ [4]. Bradybaenidae appeared to be an outlier among molluscs and most other invertebrates which exhibit much smaller differences between $\mu$_m_ and $\mu$_n_ (i.e., generally less than ten-fold differences). Results from my analysis of 121 mtDNA and nuDNA datasets of various gastropod clades show that Bradybaenidae is not an outlier within Gastropoda. Indeed, groups of species from most of the subclasses examined exhibited values of $\mu$_m_/$\mu$_n_ that are relatively large (i.e., > 20). Moreover, several groups have values of $\mu$_m_/$\mu$_n_ that exceed the value previously estimated for Bradybaenidae (Fig. 1). Based on analysis of patterns of divergence of mtDNA and nuDNA, differences in $\mu$_m_/$\mu$_n_ among gastropod taxa appear to reflect differences in $\mu$_n_ and not $\mu$_m_ (Fig. 2). Nonetheless, given that datasets with exceptionally large values of $\mu$_m_/$\mu$_n_ include low levels of taxonomic breadth, the relatively low values of $\mu$_n_ that were estimated for these datasets may be small because they contain recently diverged taxa that exhibit very few if any fixed differences at nuDNA.

Datasets from 11 groups are identified as outliers given that they exhibit values of $\mu$_m_/$\mu$_n_ that are considerably larger (N = 10) or smaller (N = 1) than values from related groups (Fig. 1, Table 2). The species contained in the ten datasets that have relatively large values of $\mu$_m_/$\mu$_n_ exclusively represent members of single genera that have radiated recently [15–23]. For example, the *Conus* dataset that represents an outlier ($\mu$_m_/$\mu$_n_ = 56.7) includes 44 members of the Cape Verde species flock, a group of species that may have radiated explosively during the past few million years [15, 24, 25]. An additional dataset that contains members of the superfamily Conoidea, including six *Conus* species (but not any from the Cape Verde species flock), represented an outlier because its value of $\mu$_m_/$\mu$_n_ (5.9) was less than those of related taxa (Fig. 1). Excluding all but these six *Conus* species yields a value of 9.0 for $\mu$_m_/$\mu$_n_. Although this value is larger than the value exhibited by members of the entire superfamily, it is still much less than $\mu$_m_/$\mu$_n_ of the Cape Verde *Conus* dataset. Moreover, another dataset that includes sequences of three other *Conus* species (and none from the Cape Verde species flock) also has a relatively small value of $\mu$_m_/$\mu$_n_ (11.2). Hence, while some *Conus* species exhibit a relatively modest value of $\mu$_m_/$\mu$_n_, species from Cape Verde species that underwent a recent radiation show an exceptionally large one.

Datasets with relatively large values of $\mu$_m_/$\mu$_n_ show smaller values of $\mu$_n_ (i.e., TBL of nuDNA gene trees) relative to the number of species included in tree compared to datasets with relatively small values of $\mu$_m_/$\mu$_n_ (Fig. 2). These results suggest that differences in $\mu$_m_/$\mu$_n_ among groups reflect differences in $\mu$_n_. Given that (i) all of the datasets that exhibit depressed values of $\mu$_n_ include low levels of taxonomic breadth (i.e., only include members of single genera or families) (Fig. 2) and (ii) datasets with little breadth exhibit larger values of $\mu$_m_/$\mu$_n_ than those with high levels of taxonomic breadth (i.e., include members of superfamilies and higher taxonomic categories) (Fig. 3), $\mu$_n_ appears to be associated with the taxonomic breadth of species included in datasets. Recently diverged taxa may be more likely to exhibit elevated values of $\mu$_m_/$\mu$_n_ because they show only very few if any fixed differences at nuDNA. Otherwise, some other processes or sampling artifacts may be responsible for depressing estimates of $\mu$_n_ in recently radiated taxa. The Bradybaenidae data contain sequences of many recently diverged species of several genera that do not show reciprocal monophyly in molecular phylogenies [12]. Furthermore, the largest value of $\mu$_m_/$\mu$_n_ that was reported in [4] (79.2) is for a group of recently diverged amphibian species (genus *Bufo*) [26]. While, it was hypothesized that the extreme value estimated for this group may be due to sampling error related to the small sample size of the datasets examined [4], the value instead may have been overestimated owing to the recent divergence of the species included in the dataset.

Although most of the datasets examined included coding regions of sequences of COI for the mitochondrial gene and H3 for the nuclear gene, four of the outlier datasets included sequences of coding regions of the mitochondrial gene cytB and sequences of coding regions of three additional nuclear genes (a megalin-like lipoprotein gene, adenine nucleotide transferase gene, and H4). Although it will be important to perform broader surveys of genes and gene regions (e.g., introns and intergenic regions) to further validate this pattern, it is not limited to the same mitochondrial and nuclear gene pairs and hence appears to be reasonably robust to gene sampling.

## Conclusions

Members of Gastropoda appear to show considerable variation in $\mu$_m_ and $\mu$_n_, but overall tend to exhibit lower values of $\mu$_m_/$\mu$_n_ than vertebrates [4]. Nonetheless, some of the values reported herein may reflect overestimates of $\mu$_m_/$\mu$_n_ due to the inclusion of species that show low levels of total molecular divergence at nuDNA possibly due to their recent divergence. Although comparing TBL of mtDNA and nuDNA gene trees is an effective means for determining relationships among $\mu$_m_ and $\mu$_n_, the approach may give overestimates of $\mu$_m_/$\mu$_n_ when datasets include a number of recently diverged species. Nonetheless, for groups in which fossil calibrations are not available, estimating $\mu$_m_/$\mu$_n_ could be useful for identifying clades that have radiated recently.

## Methods

### Datasets

To estimate $\mu$_m_/$\mu$_n_, I gathered mtDNA and nuDNA sequence data that were uploaded to GenBank as ‘PopSets’ or collections of sequence data (as opposed to individual sequence submissions). I utilized this strategy in an effort to ensure that different authors’ views on the identity of species did not affect estimates of $\mu$_m_/$\mu$_n_. I searched the NCBI PopSet database (https://www.ncbi.nlm.nih.gov/popset) using the term “Gastropoda [Organism]” in the search field (accessed on 18-May-2020). I downloaded search results as an XML file and parsed the data to extract various information such as PopSet title, author(s), and unique identifier; publication info; taxa represented in the PopSet; and gene name, gene source (i.e., mtDNA or nuDNA), and number of sequences. I then sorted the resultant data by gene source and PopSet author(s) to identify PopSets from the same author(s) that included both mtDNA and nuDNA sequences from at least three gastropod species. I selected PopSets that exclusively included intron or coding regions (but not both) and for which sequence data were available for both mtDNA and nuDNA. The final list of prospective PopSets included all but two of the gastropod datasets that were examined in [4]; these latter datasets included sequences of cytochrome oxidase subunit I (COI), a mtDNA gene, and histone H3 (H3), a nuDNA gene of Bradybaenidae [12] and Aglajidae [13], a member of the order Cephalaspidea.

I downloaded fasta files of PopSets or individual sequences (i.e., for the two datasets that were examined in [4] but not uploaded as PopSets) from ‘PopSet’ or ‘Nucleotide’ databases at NCBI (https://www.ncbi.nlm.nih.gov/). I aligned each set of sequences using MUSCLE [27] in Seqotron v1.0.1 [28]. I evaluated sequence datasets by eye in Seqotron to ensure that alignments were robust. This included adjustments of out of frame insertions so that they occurred in the proper reading frame and elimination of ends of sequences that appeared to be misaligned (possibly due to base call errors) because they contained insertions that affected reading frames (i.e., did not occur in multiples of three). I then compared species and individuals present in the corresponding alignments of mtDNA and nuDNA sequence data and removed species and individuals from one alignment if they were not present in the other. I also eliminated all but one representative of each species in alignments and retained sequences of individuals that were represented in both datasets and/or that were the most complete; in cases where sequences from more than one individual satisfied these criteria, I retained the individual that was listed first.

I utilized taxonomy information presented in the PopSet or GenBank files to specify the genera, families, superfamilies and higher level taxonomic categories of species included in datasets. I then reconciled this information with the hierarchical classification of gastropods presented in MolluscaBase [29].

### Sequence analyses

Total branch lengths (TBL) of mtDNA and nuDNA gene trees at putative neutral sites can be used to estimate relative differences in $\mu$_m_ and $\mu$_n_ based on calculation of the ratio $\mu$_m_/$\mu$_n_ [4]. As described in [4], total molecular divergence (i.e., TBL of gene trees) at neutral sites is a function of neutral mutation rates and divergence times [30, 31]. Given that divergence times of species represented in mtDNA and nuDNA gene trees should be the same, the ratio of the TBL of these trees (at neutral sites) provides an estimate of $\mu$_m_/$\mu$_n_ [4]. I used estimates of divergence (i.e., TBL) at third codon positions for coding regions (as implemented in [4]) and all positions of intron regions as a proxy for neutral divergence.

I used MEGAX v.10.1.8 [14] to construct gene trees and estimate branch lengths of individual datasets. I constructed individual phylograms for each locus to limit the effect of having discordant gene trees that could result in overestimates of TBL due to incomplete lineage sorting (see [4]). I specified the genetic code and examined alignments to set the appropriate codon start position. I reconstructed gene trees using the General Time Reversible model with maximum likelihood. I eliminated sites that were not defined in 80% of sequences; otherwise, all other positions were utilized in tree building. I examined gene trees in MEGA to ensure that the phylogenies did not contain any long branches that could be due to any alignment errors. I then used maximum likelihood to estimate the TBL of gene trees at third positions of codons of coding regions or all positions of intron sequences.

I performed all statistical tests in R [32]. I compared values of $\mu$_m_/$\mu$_n_ for sequence datasets that included species from the following higher level taxonomic groups: the orders Architaenioglossa, Littorinimorpha, and Neogastropoda, and the superfamilies Abyssochrysoidea and Cerithioidea from the subclass Caenogastropoda; the orders Nudibranchia, Pleurobranchida, Aplysiida, Cephalaspidea, Ellobiida, and Runcinida, suborders Achatinina and Helicina, and superorder Hygrophila of the subclass Heterobranchia; the subclass Patellogastropoda; and the orders Lepetellida and Trochida from the subclass Vetigastropoda. I used average values of $\mu$_m_/$\mu$_n_ for datasets that included more than one mtDNA or nuDNA gene region. I compared $\mu$_m_/$\mu$_n_ (using log-transformed values) among these groups with ANOVA and used a Tukey test to identify groups with significant differences in $\mu$_m_/$\mu$_n_. I utilized boxplots to visualize patterns of variation of $\mu$_m_/$\mu$_n_ among and within gastropod taxa and identify outlier datasets.

To evaluate whether differences in $\mu$_m_/$\mu$_n_ ratios reflect relative increases in $\mu$_m_ or decreases in $\mu$_n_, I compared TBL of mtDNA and nuDNA gene trees to the number of species included in these trees. Measures of TBL should increase proportionally to the number of species examined [33]. I specifically compared TBL of mtDNA and nuDNA gene trees among datasets that exhibited different relative values of $\mu$_m_/$\mu$_n_ (i.e., less than and greater than 20) and determined levels of significance with an ANOVA based on comparison of log-transformed values of TBL that were standardized to the number of species included in the tree.

If values of $\mu$_m_/$\mu$_n_ are overestimated because they include recently diverged species, $\mu$_m_/$\mu$_n_ values that are calculated from datasets that include little taxonomic breadth (e.g., those including representatives of genera and families) will be greater than values from datasets that include more taxonomic breadth (e.g., those including members of superfamilies, suborders, etc.). To determine if this is the case, I compared estimates of $\mu$_m_/$\mu$_n_ among datasets that include different levels of taxonomic breadth. While some PopSets only included members of single genera, others included various members of families, superfamilies, suborders, orders, and subclasses. I utilized an ANOVA to compare log-transformed values of $\mu$_m_/$\mu$_n_ among datasets representing genera, families, superfamilies and combined higher taxonomic categories; I used a Tukey test to determine which samples exhibit significantly different values. I also utilized boxplots to visualize patterns of variation among datasets that included different levels of taxonomic breadth.

## Supplementary Information

**Additional file 1: Table S1.** Information on PopSet data analyzed in this study.

## Data Availability

The data underlying this article are available in the GenBank PopSet database (https://www.ncbi.nlm.nih.gov/popset). UID numbers of PopSets and additional information on data analyzed are available in Additional file 1: Table S1.

## Abbreviations

ANT: Adenine nucleotide translocase
COI: Cytochrome oxidase subunit I
COII: Cytochrome oxidase subunit II
EF1α: Elongation factor 1-alpha
GTR: General Time Reversible
H3: Histone H3
ITS1: Internal transcribed space 1
ITS2: Internal transcribed space 2
Mlp: Megalin-like lipoprotein
mtDNA: Mitochondrial deoxyribonucleic acid
NADH: Nicotinamide adenine dinucleotide
nuDNA: Nuclear deoxyribonucleic acid
TBL: Total branch length
*μ*_m_: Mutation rates of mitochondrial deoxyribonucleic acid
*μ*_n_: Mutation rates of nuclear deoxyribonucleic acid
